# Overcoming apoptosis resistance in high risk acute lymphoblastic leukemia by SMAC mimetics in a preclinical ALL xenograft model *in vivo*

**DOI:** 10.1186/2194-7791-1-S1-A22

**Published:** 2014-09-11

**Authors:** Melanie Schirmer, Manon Queudeville, Luca Trentin, Sarah Mirjam Eckhoff, Lüder Hinrich Meyer, Klaus-Michael Debatin

**Affiliations:** Department of Pediatrics and Adolescent Medicine, University Medical Center Ulm, Ulm, Germany

Defects in cell death signaling e.g. overexpression of “Inhibitor of Apoptosis” (IAP) proteins are associated with poor prognosis and might be one reason for treatment failure and relapse of acute leukemia. Therefore, IAP antagonists, so called SMAC mimetics (SMs), provide a promising novel treatment strategy for pediatric ALL.

In this study we investigated the effects of the small molecule SM BV6 on 42 primary ALL samples. Intriguingly, 70% of all individual patient-derived leukemias showed cell death induction after BV6 treatment in a TNF-α dependent manner. Previously, we described that rapid engraftment of ALL cells in NOD/SCID mice (short Time To Leukemia, TTL^short^) is associated with deficient apoptosis signaling in ALL cells and indicative for early patient relapse. Importantly, ALL samples with a TTL^short^/early relapse phenotype showed activation of the constitutive deficient apoptosis signaling pathway upon BV6-treatment, demonstrating that SMs induce apoptosis signaling in former apoptosis resistant primary ALL cells.

We further evaluated the *in vivo* efficacy of BV6 on high-risk ALL using our NOD/SCID/huALL xenograft model in a preclinical setting. Most interestingly, a profound reduction of tumor load and prolonged survival of animals was observed upon BV6 *in vivo* treatment alone which was even more pronounced in combination with multidrug chemotherapy. Most importantly, concomitant *in vivo* therapy with Etanercept revoked the cell death inducing effect of BV6, indicating that BV6 induced apoptosis involves signaling via TNF-α and thereby provides a potential biomarker for the identification of patients who would benefit from SM treatment.

Taken together, we show that the small molecule SM BV6 induces cell death via a TNF-α loop *ex vivo* and *in vivo* in primary patient-derived ALL. Moreover, BV6 is able to overcome apoptosis deficiency of high-risk ALL leading to prolonged *in vivo* survival in a preclinical therapy model of patient-derived ALL xenograft ALL.

